# Comprehensive Genotyping in Two Homogeneous Graves' Disease Samples Reveals Major and Novel HLA Association Alleles

**DOI:** 10.1371/journal.pone.0016635

**Published:** 2011-01-28

**Authors:** Pei-Lung Chen, Cathy Shen-Jang Fann, Chen-Chung Chu, Chien-Ching Chang, Su-Wei Chang, Hsin-Yi Hsieh, Marie Lin, Wei-Shiung Yang, Tien-Chun Chang

**Affiliations:** 1 Department of Medical Genetics, National Taiwan University Hospital, Taipei, Taiwan; 2 Department of Internal Medicine, National Taiwan University Hospital, Taipei, Taiwan; 3 Institute of Biomedical Sciences, Academia Sinica, Taipei, Taiwan; 4 Institute of Public Health, National Yang-Ming University, Taipei, Taiwan; 5 Transfusion Medicine Laboratory, Medical Research Department, Mackay Memorial Hospital, Taipei, Taiwan; 6 Graduate Institute of Clinical Medicine, College of Medicine, National Taiwan University, Taipei, Taiwan; 7 Department of Internal Medicine, College of Medicine, National Taiwan University, Taipei, Taiwan; La Jolla Institute of Allergy and Immunology, United States of America

## Abstract

**Background:**

Graves' disease (GD) is the leading cause of hyperthyroidism and thyroid eye disease inherited as a complex trait. Although geoepidemiology studies showed relatively higher prevalence of GD in Asians than in Caucasians, previous genetic studies were contradictory concerning whether and/or which human leukocyte antigen (HLA) alleles are associated with GD in Asians.

**Methodology/Principal Findings:**

We conducted a case-control association study (499 unrelated GD cases and 504 controls) and a replication in an independent family sample (419 GD individuals and their 282 relatives in 165 families). To minimize genetic and phenotypic heterogeneity, we included only ethnic Chinese Han population in Taiwan and excluded subjects with hypothyroidism. We performed direct and comprehensive genotyping of six classical HLA loci (*HLA-A*, *-B*, *-C*, *-DPB1*, *-DQB1* and *-DRB1*) to 4-digit resolution. Combining the data of two sample populations, we found that B*46:01 (odds ratio under dominant model [OR]  = 1.33, Bonferroni corrected combined *P* [*P_Bc_*]  = 1.17×10^−2^), DPB1*05:01 (OR  = 2.34, *P_Bc_* = 2.58×10^−10^), DQB1*03:02 (OR  = 0.62, *P_Bc_*  = 1.97×10^−2^), DRB1*15:01 (OR  = 1.68, *P_Bc_* = 1.22×10^−2^) and DRB1*16:02 (OR  = 2.63, *P_Bc_*  = 1.46×10^−5^) were associated with GD. HLA-DPB1*05:01 is the major gene of GD in our population and singly accounts for 48.4% of population-attributable risk.

**Conclusions/Significance:**

These GD-associated alleles we identified in ethnic Chinese Hans, and those identified in other Asian studies, are totally distinct from the known associated alleles in Caucasians. Identification of population-specific association alleles is the critical first step for individualized medicine. Furthermore, comparison between different susceptibility/protective alleles across populations could facilitate generation of novel hypothesis about GD pathophysiology and indicate a new direction for future investigation.

## Introduction

Graves disease (GD, [MIM 27500], http://www.ncbi.nlm.nih.gov/Omim/) is the leading cause of hyperthyroidism and thyroid eye disease, manifested with diffuse goiter, hyperthyroidism, thyroid-specific auto-antibodies, with/without ophthalmopathy and/or dermopathy [1]. Its prevalence in general population is around 1.0–1.6%, more common in females [2], [3]. The etiology of GD is multifactorial, with considerable genetic influence [1], evidenced by family clustering (λ_sister_ between 8 and 15) [4] and a higher concordance rate in monozygotic twins (0.35) than in dizygotic twins (0.03) [5]. The genetic contribution to GD was estimated as high as 79% [5]. Although geoepidemiology studies show relatively higher prevalence of GD in Asians than in Caucasians [6], whether/what genetic factors are important for GD in Asians is not yet clear [7]–[9].

As an autoimmune disorder, the pathogenesis of GD remains elusive. Among all the methods for studying diseases pathophysiology, genetic approach has valuable capability as being both hypothesis-testing and hypothesis-generating. Linkage analysis for GD, although yielded inconsistent results across studies [8], [10]–[14], did demonstrate that the HLA region is linked to GD susceptibility in both Caucasian and Chinese Han populations according to others' [10] and our [13] studies. Association studies have been more replicable, with a few promising loci such as the HLA region, *CTLA4*, *PTPN22*, *CD40*, *FCRL3*, *CD25*, *TG* and *TSHR* [8], [9], [15], [16]. Although the HLA loci were most promising, the risk alleles identified in Caucasians (such as the HLA-DRB1*03, C*03, C*07, C*16 and the DRB1*03-DQB1*02-DQA1*05:01 haplotype) [7], [8], [11], [17] showed no associations in Asians. (It is noteworthy that throughout this manuscript we have adapted the new HLA nomenclature system [18], which was mandated to become effective since April 2010.) Instead, in studies conducted in Chinese, Japanese, Koreans and Thai, GD was reported to associate with other class I or class II alleles [8], [11], [19]–[32] (Supplemental Table S1). There has been no conclusion regarding which HLA alleles are associated with GD in Asians [11], [15]. The reports from previous studies were contradictory, at least partly because of issues related to sample sizes, sample heterogeneity (both in ethnic background and phenotype), population stratification, genotyping resolution, and extent of coverage. Direct HLA allele genotyping (instead of using nearby SNPs as surrogates) is very expensive and requires special techniques, which might explain why most previous studies only could afford small sample sizes and limited extent of coverage.

In this study, we conducted a case-control association study (499 unrelated GD cases and 504 controls) by direct and comprehensive genotyping of 6 classical HLA loci (*HLA-A*, *-B*, *-C*, *-DPB1*, *-DQB1* and *-DRB1*) to 4-digit resolution. For replication, we used an independent cohort of family samples (419 GD individuals and their 282 relatives in 165 extended families) genotyped with a different platform with the same 6-locus coverage and 4-digit resolution. We also managed to reduce heterogeneity in genetic background by including only ethnic Chinese Han individuals, and in disease phenotype (in our family samples) by excluding subjects with family history of hypothyroidism [13]. In contrast to the known associated HLA alleles in Caucasians, we found a whole distinct spectrum of associated alleles.

## Results

### HLA association tests using unrelated GD cases and controls

In the case-control association study, we observed a total of 196 HLA alleles from 6 loci (minimum: 18 alleles from *HLA-DQB1*, maximum: 60 alleles from *HLA-B*) (Supplemental Table S2). Because of limited power to detect association with rare alleles, we only tested for disease association with common alleles (with a frequency higher than 5% in either cases or controls) (*HLA-A*: 6 alleles; *HLA-B*: 4 alleles; *HLA-C*: 5 alleles; *HLA-DPB1*: 4 alleles; *HLA-DQB1*: 7 alleles and *HLA-DRB1*: 8 alleles). For the results to be robust, we reported Bonferroni corrected *P* values as our main results in the text as well as in the Tables. However, for the purpose of comprehensiveness, we also kept some nominal *P* values in certain columns of the Tables. (Please see the “Statistical analysis” section in “Materials and Methods” for details.) Of these 34 alleles tested, we found 8 alleles showing frequency difference with nominal *P* values smaller than 0.05 in the Armitage trend test, as well as in allelic test and in association test under dominant-model. However, only 4 of the 8 alleles were statistically significant after Bonferroni correction (DPB1*05:01, odds ratio under dominant model [OR]  = 2.34, Bonferroni-corrected *P* = 1.6×10^−6^; DQB1*05:02, OR  = 2.34, Bonferroni-corrected *P* = 1.5×10^−4^; DRB1*12:02, OR  = 0.51, Bonferroni-corrected *P* = 1.7×10^−2^; DRB1*16:02, OR  = 2.63, Bonferroni-corrected *P* = 5.4×10^−6^) (Table 1). Both susceptibility alleles and protective alleles were found. It is noteworthy that the alleles associated with GD in Caucasians showed either no evidence of association (DRB1*03, DQB1*02, C*07 and C*03) or were not observed in our samples (C*16) (Supplemental Table S3 and Table S4). DQA1*05:01, another allele on the risk haplotype (DRB1*03:01-DQA1*05:01-DQB1*02) in Caucasians, was not genotyped in our study. However, in Asians, DQA1*05:01 is not known to have noticeable linkage disequilibrium with any of the susceptibility alleles we reported [33].

**Table 1:** Association results (from 499 Graves' disease cases and 504 controls) of the four alleles with Bonferroni corrected *P* value smaller than 0.05.

HLA allele	Allele Frequency(cases vs. controls)	Allelic test^a^(nominal *P* value)	Genotypic test^a^(nominal *P* value)	Armitage trend test^a^ (nominal *P* value)	Dominant model^a^(nominal *P* value)	Dominant model^a^(Bonferroni corrected *P* value)
DPB1*05:01	52.6% vs. 43.5%	OR = 1.44*P = *1.0×10^−4^	3.0×10^−7^	1.0×10^−6^	OR = 2.34*P = *4.7×10^−8^	*P = *1.6×10^−6^
DQB1*05:02	16.3% vs. 9.3%	OR = 1.89*P = *2.9×10^−6^	3.0×10^−5^	1.0×10^−5^	OR = 2.00*P = *4.3×10^−6^	*P = *1.5×10^−4^
DRB1*12:02	4.7% vs. 8.6%	OR = 0.53*P = *5.6×10^−4^	1.1×10^−3^	5.6×10^−4^	OR = 0.51*P = *4.9×10^−4^	*P = *1.7×10^−2^
DRB1*16:02	10.9% vs. 4.8%	OR = 2.43*P = *3.5×10^−7^	8.9×10^−7^	1.9×10^−7^	OR = 2.63*P = *1.6×10^−7^	*P = *5.4×10^−6^

a All *P* values (except for those in the last column) reported were nominal *P* values. The study-wide significance cut-off nominal *P* value should be 0.00147 ( = 0.05/34, which is the Bonferroni correction for a total of 34 tested alleles). The statistically significance level for Bonferroni corrected *P* value (reported in the last column) should be 0.05. OR, odds ratio.

### Replication using the family-based study and other supporting evidence from previous association reports in Asians

We next tested the familial cohort for replication using a different genotyping platform. The comprehensive FBAT *P* values were summarized in Table 2 and Supplemental Table S4. We then calculated the Bonfferoni corrected *P* values of our combined case-control and family-based analysis. We found that B*46:01 (odds ratio under dominant model [OR]  = 1.33, Bonferroni corrected combined *P* [*P_Bc_*]  = 1.17×10^−2^), DPB1*05:01 (OR  = 2.34, *P_Bc_* = 2.58×10^−10^), DQB1*03:02 (OR  = 0.62, *P_Bc_* = 1.97×10^−2^), DQB1*05:02 (OR  = 1.89, *P_Bc_* = 1.60×10^−3^), DRB1*15:01 (OR  = 1.68, *P_Bc_* = 1.22×10^−2^) and DRB1*16:02 (OR  = 2.63, *P_Bc_* = 1.46×10^−5^) were associated with GD. Review of GD association studies previously conducted in Asian populations revealed that 4 (B*46:01, DPB1*05:01, DQB1*05:02 and DRB1*16:02) of our alleles were reported as risk alleles in at least two studies, and one allele (DRB1*12:02) (, which showed protective effect in our case-control study but was unable to be replicated in our family-based study), was reported as a protective allele previously (Supplemental Table S1). Again, neither our family-based association study nor the literature review showed supports for alleles associated in Caucasian populations (Supplemental Table S1 and Table S4).

**Table 2:** Replication with our family-based study and/or previous studies in Asians.

HLA allele	Case-control study under dominant model (nominal *P* value)	Family-based study under dominant model(nominal *P* value)	Combined *P* value(nominal *P* value)	Combined *P* value(Bonferroni corrected *P* value)	Positive results in previous studies in Asian populations^a^
B*46:01	4.9×10^−2^	5.5×10^−3^	3.4×10^−4^	1.2×10^−2^	Chan *et al*. 1978 [20]; Hawkins *et al*, 1985 [21]; Yeo *et al*. 1989 [22]; Dong *et al*. 1992 [23]; Inoue *et al*. 1992 [24]; Onuma *et al*. 1994 [25]; Caven *et al*. 1994 [26]; Huang *et al*. 2003 [19]; Park *et al*. 2005 [27]
DPB1*05:01	4.7×10^−8^	7.3×10^−5^	7.6×10^−12^	2.6×10^−10^	Dong *et al*. 1992 [23]; Onuma *et al*. 1994 [25]; Takahashi *et al*. 2006 [28].
DQB1*03:02	8.9×10^−3^ (Pro)^b^	4.7×10^−2^ (Pro)	5.8×10^−4^ (Pro)	2.0×10^−2^	Nil
DQB1*05:02 c	4.3×10^−6^	8.0×10^−1^ (Pro)	4.7×10^−5^	1.6×10^−3^	Park *et al*. 2005 [27]; Wongsurawat *et al*. 2006 [29]
DRB1*15:01	2.8×10^−3^	4.9×10^−2^	3.6×10^−4^	1.2×10^−2^	Nil
DRB1*16:02	1.6×10^−7^	6.5×10^−1^	4.3×10^−7^	1.5×10^−5^	Park *et al*. 2005 [27]; Wongsurawat *et al*. 2006 [29]

a The more detailed summary of previous Asian HLA-GD association studies can be found in Supplemental Table S1.

b The annotation “(Pro)” indicates “protective” effect.

c Although DQB1*05:02 got association signals from multiple independent studies, we consider these association signals were caused by the linkage disequilibrium between DQB1*05:02 and DRB1*16:02. Please see the main texts for detailed analyses.

### Dissection of individual effect of each associated allele

**Figure 1:**
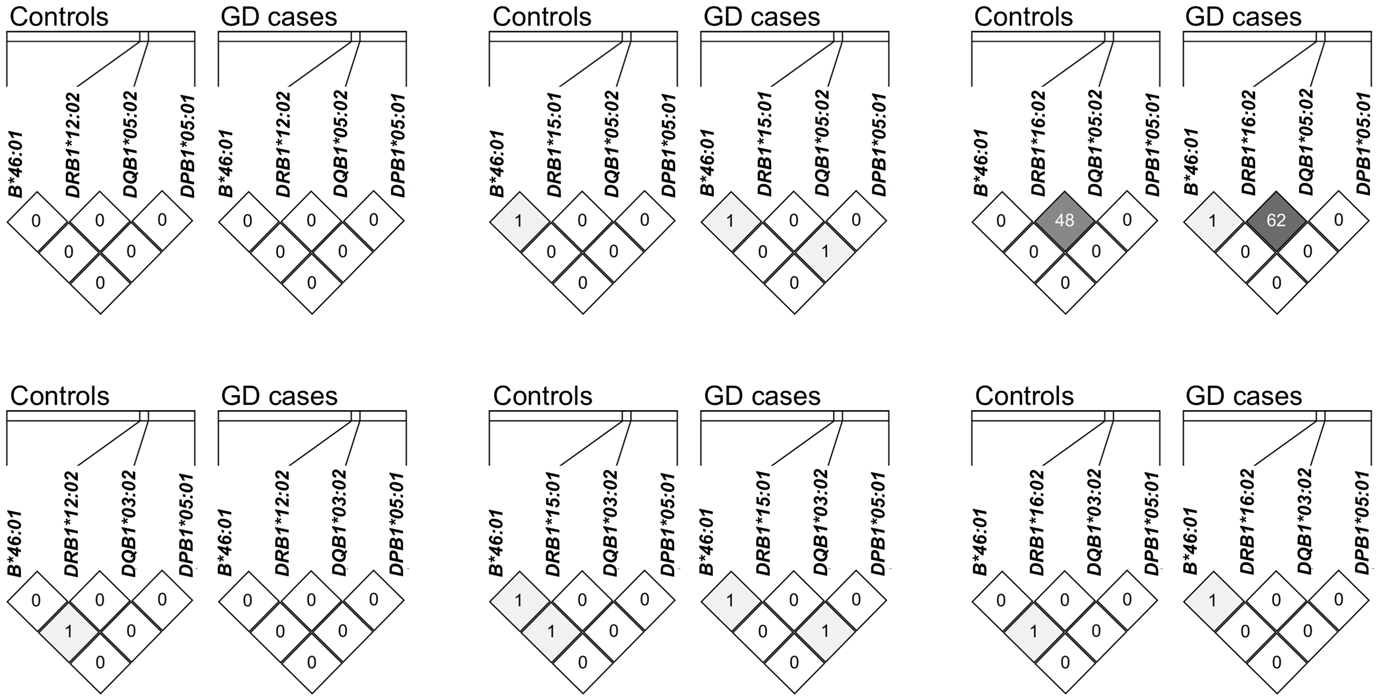
Linkage disequilibrium analysis of *HLA-B*, *HLA-DRB1*, *HLA-DQB1 *and *HLA-DPB1* alleles showing significant associations with GD. The distances between consecutive loci are approximately 1225 Kb, 81 kb and 416 Kb respectively. The r^2^ value (× 100) of any allele pair was plotted inside the corresponding cell. Except for strong linkage disequilibrium (r^2^ = 0.48 in controls, r^2^ = 0.62 in cases) between DRB1*16:02 and DQB1*05:02, in general the r^2^ value between other alleles was quite low.

It is well known that there are certain extended haplotypes across classical HLA loci [33]. In order to know if some of the observed associations represented the same association signal caused by linkage disequilibrium (LD), we therefore analyzed the LD between these 7 alleles (of 4 loci) with association signals (B*46:01 of the *HLA-B* locus; DRB1*12:02, DRB1*15:01 and DRB1*16:02 of the *HLA-DRB1* locus; DQB1*03:02 and DQB1*05:02 of the *HLA-DQB1* locus; DPB1*05:01 of the *HLA-DPB1* locus). The pairwise r^2^ values of almost all allele pairs were <0.02 (Figure 1), indicating that most of the association signals were independent from each other. The only exception was the LD between DQB1*05:02 and DRB1*16:02 (cases: r^2^ = 0.62; controls: r^2^ = 0.48) (Figure 1). Further analysis showed that all DRB1*16:02 alleles were on the DQB1*05:02-DRB1*16:02 haplotype, and the haplotype frequency was 4.77% in controls and 10.89% in GD cases (*P* = 3.50×10^−7^). On the other hand, those chromosomes containing DQB1*05:02 but not carrying this haplotype showed similar frequencies in controls (4.57%) and cases (5.44%) (*P* = 0.383). Therefore, the observed association of DQB1*05:02 is secondary to its LD with DRB1*16:02, and by itself DQB1*05:02 did not confer independent susceptibility.

### HLA-DPB1*05:01 confers susceptibility through a dominant mode of effect

It is not clear previously whether HLA alleles confer susceptibility/protective effect to GD through a dominant, additive or recessive mode. We found that the subjects with one DPB1*05:01 allele (OR  = 2.37, 95% confidence interval [CI]  = 1.72–3.62) and those with two alleles (OR  = 2.25, CI  = 1.51–3.34) had similar OR compared to the individuals with zero allele, suggesting that HLA-DPB1*05:01 confers susceptibility through a dominant mode of inheritance (Table 3). For other alleles, the allele frequencies were not high enough for us to perform similar analyses. It is noteworthy that DPB1*05:01 showed deviation from Hardy-Weinberg (HW) equilibrium in GD cases (*P* = 1.6×10^−10^) but not in controls (*P* = 0.14) (Table 3). There were more heterozygotes in the unrelated GD cases than expected under HW equilibrium, which is compatible with the dominant mode. The DPB1*05:01 genotypes from the family sample also showed similar HW disequilibrium pattern with increased heterozygotes in probands (*P* = 0.0059), but not in family founders (*P* = 0.67) (Table 3).

**Table 3:** Analysis of DPB1*05:01 genotype distribution and odds ratio.

	X/X^a^	*05:01*/X^a^	*05:01*/*05:01* a	Hardy-Weinberg equilibrium test
Founders(Family samples)	66 (23.1%)^b^	146 (51.2%)	73 (25.6%)	*P* = 0.67
Probands(Family samples)	24 (14.5%)	99 (60.0%)	42 (25.5%)	*P* = 5.9×10^−3^
Controls(Unrelated samples)	151 (30.0%)	264 (52.5%)	88 (17.5%)	*P* = 0.14
Cases(Unrelated samples)	77 (15.5%)	319 (64.2%)	101 (20.3%)	*P* = 1.6×10^−10^
Odds ratio^c^(X/X as reference)	Reference	2.37CI (1.72–3.26)	2.25CI (1.51–3.34)	
Odds ratio(X/0501 as reference)	0.42CI (0.31–0.58)	Reference	0.95CI (0.68–1.32)	

a “X” indicates “any *DPB1* allele except for DPB1*05:01”. Therefore X/X means zero DPB1*05:01 allele, *05:01*/X means one DPB1*05:01 allele and *05:01*/*05:01* means two DPB1*05:01 alleles.

b For DPB1*05:01 genotype distribution of 4 different groups of individuals (4 different rows), each cell is presented as count of individuals of that specific genotype followed by the row percentage (inside the parenthesis).

c Odds ratio is calculated based on unrelated cases and unrelated controls. CI, 95% confidence interval.

### Sizeable population-attributable risk percentage of these HLA alleles

**Figure 2:**
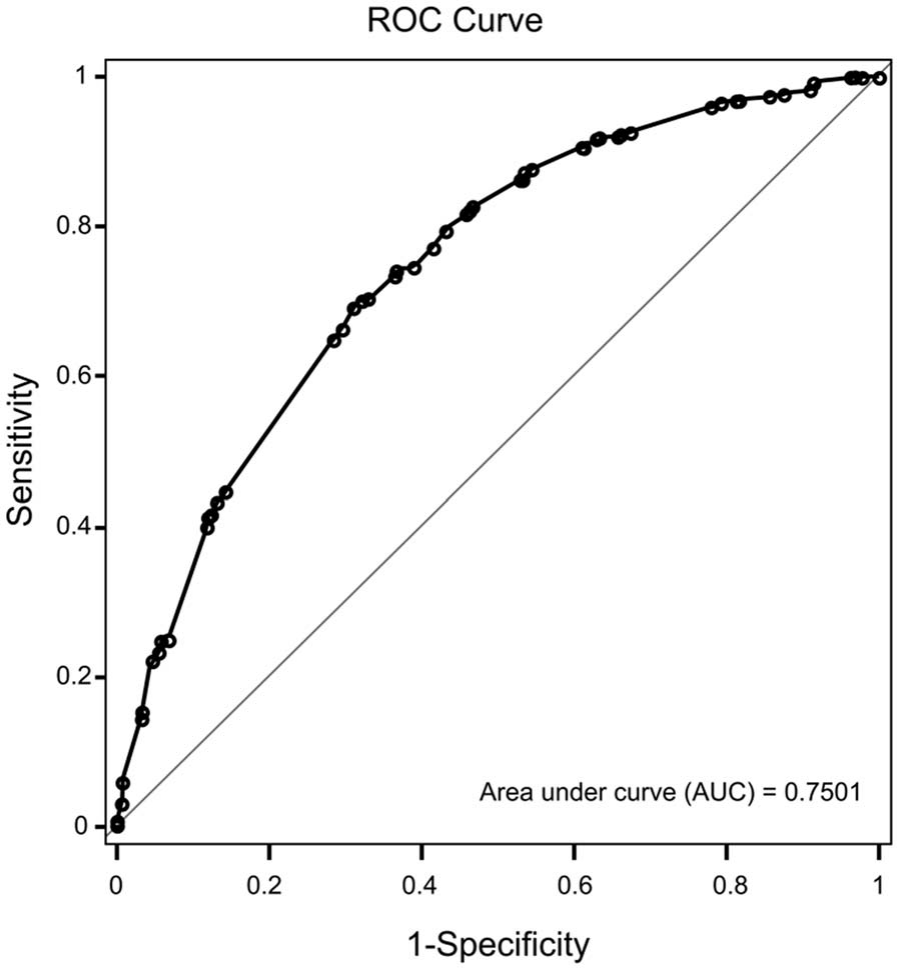
Receiver operating characteristics (ROC) curve for the logistic regression model. Disease  =  −3.6802+0.4487×B*46:01+0.8883×DPB1*05:01 – 0.3494×DQB1*03:02 – 0.492×DRB1*12:02+0.8388×DRB1*15:01+1.0727×DRB1*16:02+1.5865×**Female.** The logistic regression model was built based on the data from our unrelated case-control study individuals. Genotypes were coded following a dominant inheritance mode. The area under curve (AUC) of this ROC curve is 0.75.

These HLA alleles conferred sizeable population-attributable risk percentage (PAR%) for GD (Table 4). DPB1*05:01 singly accounts for 48.4% of population-attributable risk. We built a logistic regression model for Chinese Han population in Taiwan based on the data of these 6 alleles and gender, and the area under curve of the receiver operating characteristic (ROC) curve was 0.75 (Figure 2). Examining the PAR% (Table 4) and logistic regression models (data not shown) further supported that the association signal from DQB1*05:02 was due to its LD with DRB1*16:02.

**Table 4:** Population-attributable risk percentage of seven associated alleles and one haplotype under dominant model.

HLA allele or haplotype	Frequency of (AA + Aa)^a^	Odds ratio^b^	PAR%^c^
B*46:01	25.2%	1.33	7.7%
DPB1*05:01	70.0%	2.34	48.4%
DQB1*03:02	17.3%	0.62	−7.1%
DRB1*12:02	16.7%	0.51	−8.9%
DRB1*15:01	12.9%	1.68	8.1%
DRB1*16:02 d	9.3%	2.63	13.3%
DQB1*05:02-DRB1*16:02 d	9.3%	2.65	13.3%
DQB1*05:02 d	17.9%	2.01	15.3%

a Homozygotes or heterozygotes for the specific allele of interest. This kind of coding is to test PAR% under dominant model.

b Odds ratio under dominant model.

c PAR% would be a negative value when the allele is protective.

d The PAR% values of DQB1*05:02, DRB1*16:02 and the DQB1*05:02-DRB1*16:02 haplotype are very similar. A single susceptibility allele (most likely DRB1*16:02, please see the main text for details) is responsible for the risk, and therefore these three PAR% should only be counted once.

## Discussion

Association analysis is powerful for genetic mapping, but has been criticized for frequent spurious signals resulted from population stratification. The ways to ensure more robust results include using family-based samples and/or getting independent replications. Herein we report convincing data using both ways. Before our study, HLA-B*46 might be the only HLA allele associated with GD with good replications in Asians [8], [11], [19]. In this study, we establish the paramount role of one allele (HLA-DPB1*05:01), discover two novel associated alleles (DQB1*03:02 and DRB1*15:01), provide convincing replications of other three alleles (B*46:01, DRB1*12:02 and DRB1*16:02), and exclude independent effect of one allele (DQB1*05:02). We consider these 6 alleles to be genuine susceptibility/protective HLA alleles in our ethnic Chinese population, and probably in other Asian populations.

A recent geoepidemiology review [6] demonstrated that, unlike other autoimmune diseases (such as type 1 diabetes, multiple sclerosis and inflammatory bowel disease) which in general have higher prevalence in Caucasians, Graves' disease seems to have slightly higher prevalence in Asians. Be the relatively high prevalence of GD caused by genetic factors or environmental factors (or the interplay of both) is still an open question. However, the well-established HLA risk alleles of GD in Caucasians (HLA-DRB1*03, C*03, C*07, C*16) have either low or extremely low allele frequencies in Asians [33], [34]. The risk allele of *PTPN22*, a major autoimmune susceptibility gene of GD and several other autoimmune diseases in Caucasians, is non-polymorphic in Asians [35], [36]. Therefore, it is obvious that the genetic landscapes of GD in Asians and in Caucasians are quite different. However, even after decades of research, the major susceptibility/protective genes of GD in Asians were still unclear. Our current study establishes the major role of HLA-DPB1*05:01 (PAR%  = 48.4%), discovers two novel associated HLA alleles, and confirms three other HLA alleles. We believe that, after our current work and a careful comprehensive review of earlier GD association studies in Asians, the missing genetic “dark matter” in Asians is beginning to be observed.

Not all of the associated alleles in our case-control study were replicated in our family-based association test. Admittedly, the sample size of our family collection, although among the largest GD family collections worldwide, was still not big enough to always detect genuine association alleles with moderate effect sizes. Furthermore, due to the stochastic nature of sample collection in association study, any two independent studies (even with the same theoretical statistical power) may not detect the same association signals. In this current manuscript, for those alleles that could not be directly replicated in our family-based association test, at least the directions of effects were the same (both susceptible or both protective in our case-control study and family-based study) (Table 2), and support from previous studies in Asians could be found (also with the same directions of effects) (Table 2). The ultimate proof will rely on future association studies and/or functional assays.

Direct genotyping of classical HLA alleles (instead of using nearby SNPs as surrogates) is expensive and requires special techniques. Considering the aspects of sample size, genotyping resolution and loci coverage, to our knowledge this current study has hitherto been the most ambitious design worldwide for HLA association study with GD. While the advantages of big sample size and good genotyping resolution are self-evident, the importance of comprehensive loci coverage can not be over-emphasized. Possible linkage disequilibrium between HLA loci has been a thorny issue when researchers tried to identify the genuine locus responsible for the association signal [11], [15], [17]. We consider it crucial to examine as many classical HLA loci as possible in a single study, which may provide an opportunity to delineate the contribution of each locus. In this study, we genotyped 6 classical HLA loci (*HLA-A*, *-B*, *-C*, *-DPB1*, *-DQB1* and *-DRB1*) for all participants, a design rarely found in previous HLA-GD association studies in Asians (Supplemental Table S1) or in Caucasians [11], [15]. Because of the comprehensive locus coverage, we uncovered that the association signal from HLA-DQB1*05:02 was secondary to its LD with HLA-DRB1*16:02. After careful analysis, we reported 6 susceptibility/protective alleles, each of them representing independent association signals. We did not include *DQA1* or *DRB3,4,5* in this study, partly because of the unavailability of genotyping kits and partly because that their LD with corresponding *DQB1* or *DRB1* alleles would be too tight to be delineated.

**Figure 3:**
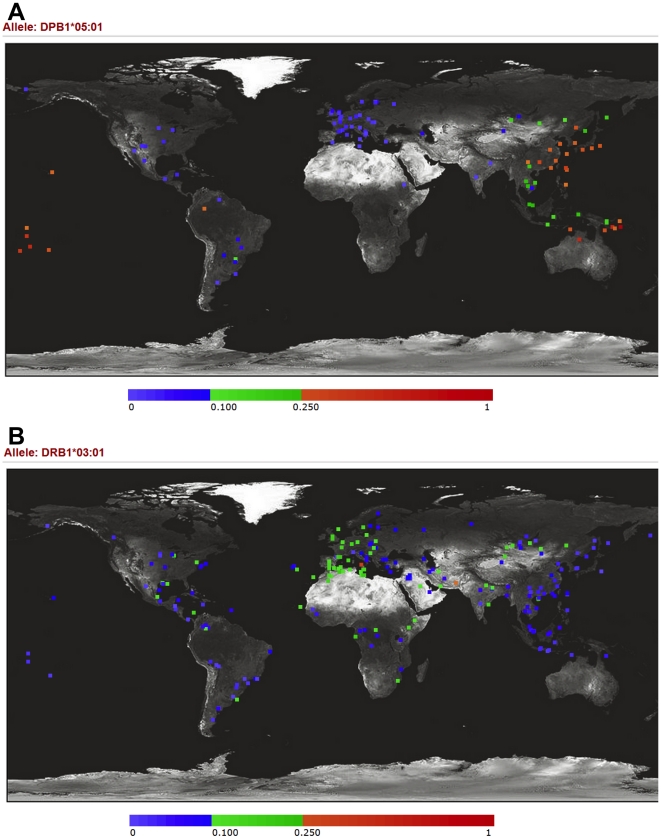
Allele frequency variations of HLA-DPB1*05:01 and HLA-DRB1*03:01 across worldwide populations. The allele frequencies (×100%) are presented with different colors, shown as the color bar below the figure. (A) HLA-DPB1*05:01, the major GD susceptibility allele we demonstrated, is much more prevalent in Asians than in Caucasians. (B) HLA-DRB1*03:01, the major GD susceptibility allele in Caucasians, has low frequencies in Asians. The data were screenshots from New Allele Frequency Database: http://www.allelefrequencies.net [33] with permission.

DPB1*05:01 has a large effect size (OR  = 2.34, under dominant model) and a very high PAR% (48.4%) in our study. It is curious why the association of DPB1*05:01 has not been addressed earlier. The association of DPB1*05:01 and GD has not been detected in Caucasians, probably because of low allele frequency (mostly <5% in Caucasians) (Figure 3) [33], [34]. In Asians, the whole *HLA-DPB1* locus was simply overlooked for more than a decade. In 1992 and 1994, three published studies [23]–[25] covered the *HLA-DPB1* locus in their study design, and actually two (Dong *et al.* [23] and Onuma *et al*. [25]) of the three reported DPB1*05:01 as a susceptibility allele. However, none of later studies incorporated the *HLA-DPB1* locus for association tests, with the only exception that in 2006 Takahashi *et al.* [28] (from the same research team as the Dong *et al.* [23] paper) reported their results (Table 1 and Supplemental Table S1). This again justifies our approach to insist on comprehensive loci coverage across the whole HLA region instead of only focusing on certain “promising” loci. Although having been overlooked in the GD research field for more than a decade, HLA-DPB1*05:01 was shown to be associated with several other immune-related phenotypes/diseases such as multiple sclerosis [37], primary biliary cirrhosis [38] and chronic hepatitis B infection [39], which, to some degree, supports that DPB1*05:01 is an HLA allele with pertinent biological significance.

It has not escaped our notice that of the four susceptibility alleles reported in this current GD study, two are well-known susceptibility alleles of multiple sclerosis (MS) (DRB1*15:01 of the conventional MS worldwide [40] and DPB1*05:01 of the opticospinal MS in Asians [37]). Some (but not all) previous studies [41] supported that GD and MS might co-occur at greater than expected rates within proband patients or their families. It would be intriguing to explore if there are common pathogenesis pathways between these two diseases.

It seems to be counterintuitive that the spectra of susceptibility/protective HLA alleles of GD are completely different between Caucasians and Asians. The main reason for this probably is the difference in allele frequencies. The most prominent susceptibility allele in Caucasians, DRB1*03:01, has a much lower frequency in Asians, ranging from <3% in Japanese and Koreans to 4–9% in Chinese (Figure 3) [33], [34] while of the six alleles we report here, four (B*46:01, DPB1*05:01, DRB1*12:02 and DRB1*16:02) have very low frequencies in Caucasians (Figure 3) [33], [34]. There have been several examples that certain susceptibility/protective alleles of genes for other autoimmune diseases varied in frequencies across populations [36], [37].

Genetic study, aside from testing existed hypothesis, has a special capability of generating new hypothesis. Difference of susceptibility/protective alleles across populations provides a great opportunity for investigating the mechanism how HLA molecules get involved in GD pathogenesis. At least partly inspired by the successful examples of the “shared epitope hypothesis” for pathogenesis of rheumatoid arthritis or type 1 diabetes mellitus [36], [42], [43], it has been postulated that arginine at position 74 of the HLA-DRB1 chain is critical for GD pathogenesis [44], mostly based on the association findings from studies conducted in Caucasians. However, the residues at position 74 of DRB1*15:01 and DRB1*16:02 reported in our association study are both alanine [45], which is the common residue at this position considered to be neutral for GD risk [44]. Further we also found susceptibility/protective alleles at class I loci, and other class II alleles. Accounting for all available evidence, we propose that the HLA region critical for GD pathogenesis is not only limited to position 74 of the DRB1 molecule. We did not find a single sequence “signature” which can explain all the associated HLA alleles identified in Caucasians and Asians. Comparison of the 3-D structure of various associated alleles and careful examination of joint effect of more than one HLA molecules might provide better hints for future study.

In summary, we report the results of our case-control and family-based GD-HLA association tests, with some strong supporting evidence from previous studies in Asians. The associated alleles are quite different from those discovered in Caucasians. HLA-DPB1*05:01 is the major gene of GD in our population, and a total of 6 susceptibility/protective alleles account for sizeable population-attributable risk. Identification of population-specific association alleles is the critical first step for individualized medicine. Furthermore, comparison between different susceptibility/protective alleles across populations could facilitate generation of novel hypothesis about GD pathophysiology and indicate a new direction for future investigation.

## Materials and Methods

### Ethics statement

The study was approved by the Institutional Review Board of National Taiwan University Hospital. Written informed consent was obtained from all GD patients and their relatives who participated in this project. The population-based unrelated controls were from the “Han Chinese Cell and Genome Bank in Taiwan” [46].

### Participant enrollment and diagnosis

The diagnosis of GD was made based on the presence of biochemical hyperthyroidism together with either the presence of thyroid eye disease or a diffuse goiter and a significant titer of auto-antibodies (including anti-microsomal, anti-thyroglobulin or anti-TSH receptor antibody) as previously reported [13]. To enrich phenotypic homogeneity, (in our family collection,) families having any family member with known possible Hashimoto's thyroiditis ([MIM603372]) were not included. Furthermore, only subjects whose four grandparents were of Chinese Han origin were included in order to avoid heterogeneity in genetic background. GD patients were recruited from individuals attending the outpatient clinic of National Taiwan University Hospital or affiliated Far Eastern Polyclinics. Pedigrees were ascertained through a GD proband. All the individuals enrolled in this study were interviewed and assessed by board-certified endocrinologists. The population-based unrelated controls were from the “Han Chinese Cell and Genome Bank in Taiwan” [46]. As in other GD studies, our unrelated cases showed a higher proportion of females (82.2%) than males. The average age of unrelated GD cases was 41.9 years (s.d. = 12.4 years), and of unrelated controls was 55.7 years (s.d.  = 18.5 years).

### Genotyping

For samples in our case-control study, we determined *HLA-A*, *-B*, *-C*, *-DQB1* and *-DRB1* genotypes using the Dynal RELI SSO typing kits (Dynal biotech Ltd, Bromborough, Wirral, U.K., now part of Life Technologies, Carlsbad, CA, USA) (http://www.invitrogen.com/) according to manufacturer's instructions. Briefly, polymerase chain reactions (PCR) using locus-specific primer sets were applied to amplify both exon 2 and exon 3 of class I (*HLA-A*, *-B* and *-C*) genes or exon 2 of class II *(-DQB1* and *-DRB1*) genes. Subsequently, PCR products were hybridized with sequence-specific oligonucleotide (SSO) probes previously fixed in a linear array on a nylon membrane (*HLA-A*: 48 probes, *-B*: 61 probes, *-C*: 37 probes, *-DQB1*: 41 probes and *-DRB1*: 60 probes). We then interpreted the genotypes using the Pattern Matching program (Dynal biotech Ltd). Due to the lack of *DPB1* genotyping kit in the Dynal RELI SSO system, we genotyped *HLA-DPB1* based on a sequence-specific primer (SSP) amplification method using “Gold SSP *HLA-DPB1* High resolution Kit” (Invitrogen Corp., now part of Life Technologies, Carlsbad, CA, USA) (http://www.invitrogen.com) according the manufacturer's protocol. Briefly, forty-eight PCR reactions were performed for each DNA sample. After PCR amplification and electrophoresis, the patterns of positive amplifications were used to interpret *HLA-DPB1* genotypes with the company's UniMatch software (Invitrogen Corp.).

For samples in our family-based study, we performed genotyping for all these 6 HLA loci using a different platform, the LABType SSO kit (One lambda Inc., Canoga Park, CA, USA) (http://www.onelambda.com/), in order to prevent potential spurious association caused by the same platform-related genotyping error. Briefly, PCR products were hybridized with probes bound to fluorescently coded micro-spheres (*HLA-A*: 58/61/63 probes, *-B*: 100 probes, *-C*: 56 probes, *-DPB1*: 40 probes, *-DQB1*: 37 probes and *-DRB1*: 70 probes). Subsequently, a flow analyzer was used to identify the fluorescent intensity on each micro-sphere (LABType visual software; One lambda Inc.) and assignment of HLA genotype was obtained based on the reaction pattern.

Ambiguity, which refers to the same reaction patterns produced by several genotype combinations [47], was dealt with by assigning allele genotypes according to common alleles (allele frequency >0.01) found in Taiwanese population [48] and southern Chinese populations [33], [34] as determined in the population studies of the 13^th^ international histocompatibility workshop.

### Statistical analysis

At any HLA loci, there are multiple alleles. We followed the common practice of most HLA association studies and coded tested alleles in a 2-allele format. For example, when we performed statistic tests for HLA-B*46:01, the allele was either coded as “HLA-B*46:01” or “X” (which meant any other possible alleles at the *HLA-B* locus). Consequently, in this example, the genotype of an individual would be coded as one of the three: B*46:01/B*46:01, B*46:01/X or X/X.

For the case-control study (499 unrelated GD cases and 504 unrelated controls), we tested each of the 34 common HLA alleles (with allele frequency greater than 5%) with 1-degree-of-freedom (d.f.) allelic test, 2-d.f. genotypic test, 1-d.f. Cochran-Armitage trend test, 1-d.f. dominant logistic regression model and 1-d.f. gender-adjusted dominant logistic regression model, using PLINK [49] v1.07 (http://pngu.mgh.harvard.edu/purcell/plink/) or SAS v9.2 (http://www.sas.com). For completeness, we calculated both nominal *P* values and Bonferroni corrected *P* values. Considering that 34 alleles were tested, regardless of possible linkage disequilibrium between certain alleles, the most conservative study-wide significance cut-off nominal *P* value for Bonferroni correction should be 0.0015 ( = 0.05/34). For all of our main results, we reported Bonferroni corrected *P* values, which were nominal P values multiplied by 34, the number of measures being tested [50], [51]. Bonferroni corrected *P* values smaller than 0.05 were considered statistically significant [50], [51].

For the results to be robust, we reported Bonferroni corrected *P* values as our main results in the text as well as in the Tables. However, for the purpose of comprehensiveness, we also kept some nominal *P* values in certain columns of the Tables.

For the family study (419 GD cases and their 282 family members in 165 extended pedigrees), we applied PedCheck v1.1 to check for genotyping error under the known family structure. We then used family-based association test [52] (FBAT) v1.7.3 (http://www.biostat.harvard.edu/~fbat/default.html) for association analyses. A dominant model was chosen based on our observation that HLA alleles (at least DPB1*05:01 shown in our analysis) might exert the effect in a dominant mode. We applied the “-e” option in FBAT to produce the empirical variance and make the test robust to the presence of linkage [52].

We calculated combined *P* values (combination of our case-control study and our family-based association test) based on the method described by de Bakker *et al.* [53]. Briefly, z statistics were calculated based on the individual original *P* values, then summed up after considering the effect direction and weighting, and then converted back to get the combined *P* value. Appropriate weighting and effective sample sizes were derived from PBAT [54] and Genetic Power Calculator [55] based on the allele frequency and OR of the controls and family founders [53]. Again, nominal *P* values smaller than 0.0015 or Bonferroni corrected *P* values smaller than 0.05 were considered statistically significant.

We analyzed the linkage disequilibrium (LD) patterns between those 7 alleles with association signals (B*46:01 of the *HLA-B* locus; DRB1*12:02, DRB1*15:01 and DRB1*16:02 of the *HLA-DRB1* locus; DQB1*03:02 and DQB1*05:02 of the *HLA-DQB1* locus; DPB1*05:01 of the *HLA-DPB1* locus) using HaploView v4.1 (http://www.broadinstitute.org/haploview/haploview) and the SAS HAPLOTYPE procedure. By definition, LD is a measurement between alleles at different loci (for example, between B*46:01 and DRB1*12:02); therefore we did not try to find if alleles of the same locus (for example, DRB1*12:02 and DRB1*15:01) co-existed too often or too rarely.

We estimated the population attributable risk percentage (*PAR*%) for the susceptibility/protective genotypes using the formula [56]: 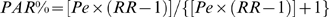
where *Pe* represents the susceptibility/protective genotype frequency (coded as the dominant-model) in the population, and *RR* represents relative risk of the risk genotype. Given the relatively low prevalence (1–1.6%) of GD [2], [3], *Pe* can be estimated based on the genotype frequencies in healthy controls, and RR can be approximated by OR of the risk genotypes [56].

## Supporting Information

Table S1 Summary of HLA association studies of Graves' disease preformed in Asian populations.
(DOC)

Table S2 A full list of HLA genotype counts and frequencies in 499 unrelated Graves' disease cases and 504 unrelated controls.
(DOC)

Table S3 Association results (from 499 Graves' disease cases and 504 controls) of all the 34 alleles with allele frequency greater than 5%.
(DOC)

Table S4 Association results of family-based association test.
(DOC)
