# Supplementary material for: Comprehensive Genotyping in Two Homogeneous Graves' Disease Samples Reveals Major and Novel HLA Association Alleles
Source: PLoS One. 2011 Jan 28;6(1):e16635. doi: 10.1371/journal.pone.0016635 (PMC3030609; doi:10.1371/journal.pone.0016635)
Supplement: Table S1 — Summary of HLA association studies of Graves' disease preformed in Asian populations. (DOC) [file pone.0016635.s001.doc]

**Table S1. Summary of HLA association studies of Graves’ disease preformed in Asian populations.**

| **Country** | **Ethnic group** | **Tested loci** | **Number of individuals**  **(cases/controls)** | **HLA allele** | **OR^a^** | **References** |
| --- | --- | --- | --- | --- | --- | --- |
| South Korea | Korean | *-A*  *-B*  *-C*  *-DR* | 128/220 | *B13*  *DR5*  *DR8* | 3.8  4.4  2.3 | Cho *et al*. 1987 [30]. |
|  |  | *-DQB1 -DRB1* | 198/200 | *DQB1*02:01*  *DQB1*05:01*  *DQB1*05:02*  *DQB1*06:01*  *DQB1*06:04*  *DRB1*01:01*  *DRB1*08:03*  *DRB1*07:01*  *DRB1*12:02*  *DRB1*13:02*  *DRB1*16:02* | 0.30  0.36  2.65  2.37  0.37  0.33  2.27  0.34  0.25  0.45  22.3 | Park *et al*. 2005 [27]. |
| Japan | Japanese | *-A*  *-B*  *-C*  *-DP*  *-DQ*  *-DR* | 76/317 | *A2*  *B46*  *C11*  *DPB1*05:01* | 2.86  2.22  2.94  5.32 | Dong *et al*^b^. 1992 [23]. |
|  |  | *-A*  *-B*  *-C*  *-DP*  *-DQ*  *-DR* | 88/186 | *B46*  *B48*  *DR8*  *DQ4* | 2.0  2.2  1.7  1.9 | Inoue *et al*. 1992 [24]. |
|  |  | *-A*  *-B*  *-C*  *-DP*  *-DQ*  *-DR* | 106/100 | *B46*  *C1*  *DPB1*05:01* | 4.8  2.8  2.2 | Onuma *et al*. 1994 [25]. |
|  |  | *-A*  *-DP* | 48/321 | *A*02:06*  *DPB1*02:01*  *DPB1*05:01*  *DPB1*13:01* | 2.02  0.51  2.61  0.25 | Takahashi *et al*^b^. 2006 [28]. |
| Hong Kong, China | Chinese Han | *-A*  *-B*  *-DQA1*  *-DQB1*  *-DR* | 97/105 | *B46*  *DR9*  *DQB1*03:03* | 2.30  2.16  3.19 | Cavan *et al*. 1994 [26]. |
|  |  | *-A*  *-B*  *-DR* | 132/110 | *B46* | 2.07 | Hawkins *et al*. 1985 [21]. |
|  |  | *-DQA1*  *-DQB1* | 67/ 51 | *DQB1*03:03* | 4.22 | Wong *et al*. 1999 [31]. |
| Singapore | Chinese Han | *-A*  *-B* | 86/238 | *B46* | 3.74 | Chan *et al*. 1978 [20]. |
|  |  | *-A*  *-B*  *-DR* | 159/330 | *B46* | 2.6 | Yeo *et al*. 1989 [22]. |
| Thailand | Thai | *-DQA1*  *-DQB1*  *-DRB1* | 124/124 | *DRB1*07*  *DRB1*16:02*  *DQA1*01:02*  *DQA1*02:01*  *DQA1*06:01*  *DQB1*05:02* | 0.25  2.91  1.91  0.25  0.36  1.94 | Wongsurawat *et al*. 2006 [29]. |
| Taiwan | Chinese Han | *-DR*  *-DQ* | 93/106 | *DR2*  *DR9*  *DR53*  *DQ1* | 2.49  2.69  1.95  2.11 | Tsai *et al*. 1989 [32]. |
|  |  | *-A*  *-B*  *-DRB1* | 236/533 | *A*02:07*  *B*27:04*  *B*46:01*  *DRB1*09:01* | 2.21  3.82  1.76  1.62 | Huang *et al*. 2003 [19]. |
|  |  | *-A*  *-B*  *-C*  *-DPB1*  *-DQB1*  *-DRB1* | 499/504 | *B*46:01*  *DPB1*05:01*  *DQB1*03:02*  *DQB1*05:02*^c^  *DRB1*12:02*  *DRB1*15:01*  *DRB1*16:02* | 1.33  2.34  0.62  2.00  0.51  1.68  2.63 | Chen *et al*. 2011  (Our current study) |

^a^ OR, odds ratio. ORs are listed here based on the way (either allelic OR or dominant-mode OR) that each individual study presented their data.

^b^ The Takahashi *et al.* [28] study was the follow-up study of the Dong *et al.* [23] study. In this table we only list the number (= 48) of “new” GD patient collection.

^c^ Although *DQB1*05:02* got association signals from multiple independent studies, we consider these association signals were caused by the linkage disequilibrium between *DQB1*05:02* and *DRB1*16:02*. Please see the main texts for detailed analyses.
