# Supplementary material for: Comprehensive Genotyping in Two Homogeneous Graves' Disease Samples Reveals Major and Novel HLA Association Alleles
Source: PLoS One. 2011 Jan 28;6(1):e16635. doi: 10.1371/journal.pone.0016635 (PMC3030609; doi:10.1371/journal.pone.0016635)
Supplement: Table S2 — A full list of HLA genotype counts and frequencies in 499 unrelated Graves' disease cases and 504 unrelated controls. (DOC) [file pone.0016635.s002.doc]

**Table S2. A full list of HLA genotype counts and frequencies in 499 unrelated Graves’ disease cases and 504 unrelated controls.**

| **HLA allele** |  | **Cases** | |  | **Controls** | |
| --- | --- | --- | --- | --- | --- | --- |
|  |  | **Count** | **Freq^a^** |  | **Count** | **Freq** |
| *A*01:01* |  | 6 | 0.006 |  | 4 | 0.004 |
| *A*01:03* |  | 1 | 0.001 |  | 2 | 0.002 |
| *A*02:01* |  | 81 | 0.081 |  | 93 | 0.092 |
| *A*02:03* |  | 78 | 0.078 |  | 64 | 0.064 |
| *A*02:06* |  | 31 | 0.031 |  | 27 | 0.027 |
| *A*02:07* |  | 134 | 0.134 |  | 113 | 0.112 |
| *A*03:01* |  | 2 | 0.002 |  | 6 | 0.006 |
| *A*03:02* |  | 0 | 0.000 |  | 1 | 0.001 |
| *A*11:01/02* |  | 374 | 0.375 |  | 328 | 0.326 |
| *A*11:04* |  | 1 | 0.001 |  | 0 | 0.000 |
| *A*24:02* |  | 129 | 0.129 |  | 159 | 0.158 |
| *A*24:03* |  | 5 | 0.005 |  | 2 | 0.002 |
| *A*24:07* |  | 4 | 0.004 |  | 2 | 0.002 |
| *A*24:10* |  | 3 | 0.003 |  | 5 | 0.005 |
| *A*26:01* |  | 25 | 0.025 |  | 24 | 0.024 |
| *A*26:02* |  | 0 | 0.000 |  | 1 | 0.001 |
| *A*29:01* |  | 1 | 0.001 |  | 3 | 0.003 |
| *A*30:01* |  | 11 | 0.011 |  | 12 | 0.012 |
| *A*31:01* |  | 14 | 0.014 |  | 28 | 0.028 |
| *A*32:01* |  | 2 | 0.002 |  | 5 | 0.005 |
| *A*33:03* |  | 95 | 0.095 |  | 119 | 0.118 |
| *A*34:01* |  | 0 | 0.000 |  | 1 | 0.001 |
| *A*68:01* |  | 1 | 0.001 |  | 4 | 0.004 |
| *A*69:01* |  | 0 | 0.000 |  | 1 | 0.001 |
| *A*74:01* |  | 0 | 0.000 |  | 2 | 0.002 |
| *B*07:02* |  | 1 | 0.001 |  | 2 | 0.002 |
| *B*07:05* |  | 3 | 0.003 |  | 4 | 0.004 |
| *B*08:01* |  | 4 | 0.004 |  | 4 | 0.004 |
| *B*13:01* |  | 75 | 0.075 |  | 56 | 0.056 |
| *B*13:02* |  | 8 | 0.008 |  | 17 | 0.017 |
| *B*15:01* |  | 28 | 0.028 |  | 26 | 0.026 |
| *B*15:02* |  | 35 | 0.035 |  | 45 | 0.045 |
| *B*15:03* |  | 2 | 0.002 |  | 2 | 0.002 |
| *B*15:07* |  | 1 | 0.001 |  | 0 | 0.000 |
| *B*15:08* |  | 0 | 0.000 |  | 1 | 0.001 |
| *B*15:11* |  | 12 | 0.012 |  | 5 | 0.005 |
| *B*15:12* |  | 3 | 0.003 |  | 2 | 0.002 |
| *B*15:13* |  | 1 | 0.001 |  | 1 | 0.001 |
| *B*15:18* |  | 3 | 0.003 |  | 10 | 0.010 |
| *B*15:25* |  | 7 | 0.007 |  | 4 | 0.004 |
| *B*15:27* |  | 9 | 0.009 |  | 12 | 0.012 |
| *B*15:32* |  | 0 | 0.000 |  | 2 | 0.002 |
| *B*15:58* |  | 2 | 0.002 |  | 0 | 0.000 |
| *B*15:68* |  | 0 | 0.000 |  | 1 | 0.001 |
| *B*18:01* |  | 1 | 0.001 |  | 1 | 0.001 |
| *B*18:02* |  | 0 | 0.000 |  | 1 | 0.001 |
| *B*27:04* |  | 19 | 0.019 |  | 26 | 0.026 |
| *B*27:05* |  | 0 | 0.000 |  | 3 | 0.003 |
| *B*27:06* |  | 0 | 0.000 |  | 1 | 0.001 |
| *B*27:07* |  | 0 | 0.000 |  | 1 | 0.001 |
| *B*35:01* |  | 34 | 0.034 |  | 20 | 0.020 |
| *B*35:02* |  | 0 | 0.000 |  | 4 | 0.004 |
| *B*35:03* |  | 0 | 0.000 |  | 1 | 0.001 |
| *B*35:05* |  | 3 | 0.003 |  | 2 | 0.002 |
| *B*37:01* |  | 3 | 0.003 |  | 3 | 0.003 |
| *B*38:02* |  | 46 | 0.046 |  | 33 | 0.033 |
| *B*39:01* |  | 14 | 0.014 |  | 25 | 0.025 |
| *B*40:01* |  | 219 | 0.220 |  | 213 | 0.211 |
| *B*40:02* |  | 7 | 0.007 |  | 15 | 0.015 |
| *B*40:03* |  | 0 | 0.000 |  | 1 | 0.001 |
| *B*40:06* |  | 21 | 0.021 |  | 12 | 0.012 |
| *B*40:40* |  | 0 | 0.000 |  | 1 | 0.001 |
| *B*44:02* |  | 2 | 0.002 |  | 2 | 0.002 |
| *B*44:03* |  | 0 | 0.000 |  | 6 | 0.006 |
| *B*46:01* |  | 173 | 0.174 |  | 137 | 0.136 |
| *B*48:01* |  | 11 | 0.011 |  | 14 | 0.014 |
| *B*48:03* |  | 1 | 0.001 |  | 2 | 0.002 |
| *B*51:01* |  | 40 | 0.040 |  | 44 | 0.044 |
| *B*51:02* |  | 16 | 0.016 |  | 16 | 0.016 |
| *B*51:07* |  | 0 | 0.000 |  | 1 | 0.001 |
| *B*52:01* |  | 11 | 0.011 |  | 10 | 0.010 |
| *B*54:01* |  | 29 | 0.029 |  | 42 | 0.042 |
| *B*55:02* |  | 20 | 0.020 |  | 35 | 0.035 |
| *B*55:04* |  | 1 | 0.001 |  | 2 | 0.002 |
| *B*55:07* |  | 1 | 0.001 |  | 0 | 0.000 |
| *B*56:01* |  | 7 | 0.007 |  | 6 | 0.006 |
| *B*56:03* |  | 4 | 0.004 |  | 5 | 0.005 |
| *B*56:04* |  | 2 | 0.002 |  | 3 | 0.003 |
| *B*57:01* |  | 0 | 0.000 |  | 3 | 0.003 |
| *B*58:01* |  | 113 | 0.114 |  | 107 | 0.106 |
| *B*59:01* |  | 0 | 0.000 |  | 1 | 0.001 |
| *B*67:01* |  | 2 | 0.002 |  | 10 | 0.010 |
| *B*81:02* |  | 1 | 0.001 |  | 2 | 0.002 |
| *C*01:02* |  | 224 | 0.225 |  | 214 | 0.212 |
| *C*01:03* |  | 3 | 0.003 |  | 2 | 0.002 |
| *C*02:02* |  | 0 | 0.000 |  | 2 | 0.002 |
| *C*03:01* |  | 1 | 0.001 |  | 0 | 0.000 |
| *C*03:02* |  | 105 | 0.106 |  | 106 | 0.105 |
| *C*03:03* |  | 50 | 0.050 |  | 42 | 0.042 |
| *C*03:04* |  | 134 | 0.135 |  | 117 | 0.116 |
| *C*03:04/03* |  | 7 | 0.007 |  | 6 | 0.006 |
| *C*03:17* |  | 0 | 0.000 |  | 1 | 0.001 |
| *C*04:01* |  | 31 | 0.031 |  | 47 | 0.047 |
| *C*04:03* |  | 16 | 0.016 |  | 19 | 0.019 |
| *C*04:06* |  | 1 | 0.001 |  | 0 | 0.000 |
| *C*05:01* |  | 1 | 0.001 |  | 1 | 0.001 |
| *C*06:01* |  | 0 | 0.000 |  | 1 | 0.001 |
| *C*06:02* |  | 11 | 0.011 |  | 20 | 0.020 |
| *C*07:01* |  | 3 | 0.003 |  | 2 | 0.002 |
| *C*07:02* |  | 212 | 0.213 |  | 203 | 0.201 |
| *C*07:04* |  | 4 | 0.004 |  | 12 | 0.012 |
| *C*08:01* |  | 70 | 0.070 |  | 82 | 0.081 |
| *C*12:02* |  | 34 | 0.034 |  | 40 | 0.040 |
| *C*12:03* |  | 10 | 0.010 |  | 12 | 0.012 |
| *C*14:02* |  | 32 | 0.032 |  | 33 | 0.033 |
| *C*15:02* |  | 39 | 0.039 |  | 41 | 0.041 |
| *C*15:02/05* |  | 2 | 0.002 |  | 0 | 0.000 |
| *C*15:05* |  | 1 | 0.001 |  | 4 | 0.004 |
| *C*16:02* |  | 2 | 0.002 |  | 0 | 0.000 |
| *C*16:04* |  | 1 | 0.001 |  | 1 | 0.001 |
| *DPB1*01:01* |  | 0 | 0.000 |  | 1 | 0.001 |
| *DPB1*02:01* |  | 142 | 0.147 |  | 173 | 0.172 |
| *DPB1*02:02* |  | 69 | 0.072 |  | 85 | 0.085 |
| *DPB1*03:01/104:01* |  | 42 | 0.044 |  | 54 | 0.054 |
| *DPB1*04:01* |  | 80 | 0.083 |  | 81 | 0.081 |
| *DPB1*04:02* |  | 19 | 0.020 |  | 16 | 0.016 |
| *DPB1*05:01* |  | 507 | 0.526 |  | 440 | 0.437 |
| *DPB1*08:01* |  | 2 | 0.002 |  | 0 | 0.000 |
| *DPB1*09:01* |  | 9 | 0.009 |  | 16 | 0.016 |
| *DPB1*13:01* |  | 37 | 0.038 |  | 52 | 0.052 |
| *DPB1*14:01* |  | 18 | 0.019 |  | 23 | 0.023 |
| *DPB1*16:01* |  | 2 | 0.002 |  | 0 | 0.000 |
| *DPB1*17:01* |  | 4 | 0.004 |  | 12 | 0.012 |
| *DPB1*19:01* |  | 9 | 0.009 |  | 13 | 0.013 |
| *DPB1*21:01* |  | 4 | 0.004 |  | 19 | 0.019 |
| *DPB1*26:01* |  | 0 | 0.000 |  | 1 | 0.001 |
| *DPB1*31:01* |  | 0 | 0.000 |  | 1 | 0.001 |
| *DPB1*48:01* |  | 0 | 0.000 |  | 1 | 0.001 |
| *DPB1*100:01* |  | 0 | 0.000 |  | 3 | 0.003 |
| *DPB1*105:01* |  | 0 | 0.000 |  | 1 | 0.001 |
| *DPB1*107:01* |  | 20 | 0.021 |  | 14 | 0.014 |
| *DQB1*02:01* |  | 102 | 0.102 |  | 86 | 0.086 |
| *DQB1*02:02* |  | 8 | 0.008 |  | 24 | 0.024 |
| *DQB1*03:01* |  | 183 | 0.183 |  | 213 | 0.212 |
| *DQB1*03:02* |  | 58 | 0.058 |  | 88 | 0.088 |
| *DQB1*03:03* |  | 162 | 0.162 |  | 172 | 0.171 |
| *DQB1*03:13* |  | 1 | 0.001 |  | 0 | 0.000 |
| *DQB1*03:14* |  | 1 | 0.001 |  | 0 | 0.000 |
| *DQB1*04:01* |  | 64 | 0.064 |  | 69 | 0.069 |
| *DQB1*04:02* |  | 21 | 0.021 |  | 15 | 0.015 |
| *DQB1*05:01* |  | 19 | 0.019 |  | 25 | 0.025 |
| *DQB1*05:02* |  | 163 | 0.163 |  | 94 | 0.093 |
| *DQB1*05:03* |  | 45 | 0.045 |  | 49 | 0.049 |
| *DQB1*06:01* |  | 105 | 0.105 |  | 108 | 0.107 |
| *DQB1*06:02* |  | 48 | 0.048 |  | 34 | 0.034 |
| *DQB1*06:03* |  | 2 | 0.002 |  | 1 | 0.001 |
| *DQB1*06:04* |  | 0 | 0.000 |  | 2 | 0.002 |
| *DQB1*06:09* |  | 14 | 0.014 |  | 22 | 0.022 |
| *DQB1*06:10* |  | 2 | 0.002 |  | 4 | 0.004 |
| *DRB1*01:01* |  | 2 | 0.002 |  | 5 | 0.005 |
| *DRB1*03:01* |  | 100 | 0.101 |  | 87 | 0.087 |
| *DRB1*04:01* |  | 2 | 0.002 |  | 6 | 0.006 |
| *DRB1*04:02* |  | 0 | 0.000 |  | 1 | 0.001 |
| *DRB1*04:03* |  | 18 | 0.018 |  | 33 | 0.032 |
| *DRB1*04:04* |  | 7 | 0.007 |  | 11 | 0.011 |
| *DRB1*04:05* |  | 67 | 0.068 |  | 77 | 0.077 |
| *DRB1*04:06* |  | 27 | 0.027 |  | 34 | 0.034 |
| *DRB1*04:07* |  | 0 | 0.000 |  | 2 | 0.002 |
| *DRB1*04:10* |  | 0 | 0.000 |  | 2 | 0.002 |
| *DRB1*07:01* |  | 10 | 0.010 |  | 28 | 0.028 |
| *DRB1*08:01* |  | 1 | 0.001 |  | 1 | 0.001 |
| *DRB1*08:02* |  | 6 | 0.006 |  | 7 | 0.007 |
| *DRB1*08:03* |  | 72 | 0.073 |  | 86 | 0.086 |
| *DRB1*08:09* |  | 14 | 0.014 |  | 3 | 0.003 |
| *DRB1*09:01* |  | 161 | 0.162 |  | 167 | 0.166 |
| *DRB1*10:01* |  | 6 | 0.006 |  | 7 | 0.007 |
| *DRB1*11:01* |  | 78 | 0.079 |  | 80 | 0.080 |
| *DRB1*11:04* |  | 4 | 0.004 |  | 2 | 0.002 |
| *DRB1*11:06* |  | 1 | 0.001 |  | 1 | 0.001 |
| *DRB1*12:01* |  | 37 | 0.037 |  | 33 | 0.033 |
| *DRB1*12:02* |  | 47 | 0.047 |  | 86 | 0.086 |
| *DRB1*12:08* |  | 0 | 0.000 |  | 1 | 0.001 |
| *DRB1*13:01* |  | 3 | 0.003 |  | 2 | 0.002 |
| *DRB1*13:02* |  | 13 | 0.013 |  | 22 | 0.022 |
| *DRB1*13:07* |  | 2 | 0.002 |  | 1 | 0.001 |
| *DRB1*13:12* |  | 11 | 0.011 |  | 6 | 0.006 |
| *DRB1*13:50* |  | 2 | 0.002 |  | 0 | 0.000 |
| *DRB1*14:01* |  | 41 | 0.041 |  | 39 | 0.039 |
| *DRB1*14:03* |  | 4 | 0.004 |  | 3 | 0.003 |
| *DRB1*14:04* |  | 2 | 0.002 |  | 6 | 0.006 |
| *DRB1*14:05* |  | 23 | 0.023 |  | 22 | 0.022 |
| *DRB1*14:10* |  | 0 | 0.000 |  | 1 | 0.001 |
| *DRB1*14:18* |  | 2 | 0.002 |  | 4 | 0.004 |
| *DRB1*14:22* |  | 0 | 0.000 |  | 1 | 0.001 |
| *DRB1*15:01* |  | 102 | 0.103 |  | 68 | 0.068 |
| *DRB1*15:02* |  | 19 | 0.019 |  | 23 | 0.023 |
| *DRB1*16:02* |  | 108 | 0.109 |  | 48 | 0.048 |

^a^ Freq, frequency.
