# Supplementary material for: Comprehensive Genotyping in Two Homogeneous Graves' Disease Samples Reveals Major and Novel HLA Association Alleles
Source: PLoS One. 2011 Jan 28;6(1):e16635. doi: 10.1371/journal.pone.0016635 (PMC3030609; doi:10.1371/journal.pone.0016635)
Supplement: Table S3 — Association results (from 499 Graves' disease cases and 504 controls) of all the 34 alleles with allele frequency greater than 5%. (DOC) [file pone.0016635.s003.doc]

**Table S3. Association results (from 499 Graves’ disease cases and 504 controls) of all the 34 alleles with allele frequency greater than 5%.**

| **HLA allele** | **Allelic OR^a^** | **Genotypic**  **Test** | **Allelic**  **Test** | **Trend**  **Test** |  | **Dominant model** | |  | **Dominant model**  **adjusted by gender** | |
| --- | --- | --- | --- | --- | --- | --- | --- | --- | --- | --- |
|  |  | ***P* value (100,000 permutation)**  **(nominal *P* value)** | | |  | **OR** | **Nominal *P* value** |  | **OR** | **Nominal *P* value** |
| *A*02:01* | 0.87 | 0.20 | 0.38 | 0.39 |  | 0.81 | 0.23 |  | 0.82 | 0.31 |
| *A*02:03* | 1.25 | 0.44 | 0.22 | 0.23 |  | 1.26 | 0.23 |  | 1.12 | 0.62 |
| *A*02:07* | 1.23 | 0.30 | 0.15 | 0.16 |  | 1.27 | 0.13 |  | 1.44 | 2.9E-2 |
| ***A*11:01/02*^b^** | **1.24** | **6.8E-2** | **2.6E-2** | **2.2E-2** |  | **1.31** | **4.0E-2** |  | **1.30** | **6.7E-2** |
| *A*24:02* | 0.79 | 0.13 | 7.3E-2 | 7.6E-2 |  | 0.75 | 5.3E-2 |  | 0.69 | 1.7E-2 |
| *A*33:03* | 0.78 | 0.20 | 8.2E-2 | 8.1E-2 |  | 0.77 | 9.9E-2 |  | 0.75 | 0.10 |
| *B*13:01* | 1.38 | 0.21 | 8.6E-2 | 8.3E-2 |  | 1.38 | 0.11 |  | 1.48 | 6.6E-2 |
| *B*40:01* | 1.05 | 0.89 | 0.66 | 0.66 |  | 1.06 | 0.65 |  | 1.01 | 0.99 |
| ***B*46:01*** | **1.34** | **6.2E-2** | **2.2E-2** | **2.5E-2** |  | **1.33** | **4.9E-2** |  | **1.47** | **1.3E-2** |
| *B*58:01* | 1.08 | 0.88 | 0.62 | 0.61 |  | 1.09 | 0.58 |  | 1.02 | 0.98 |
| *C*01:02* | 1.08 | 0.75 | 0.48 | 0.49 |  | 1.10 | 0.47 |  | 1.18 | 0.26 |
| *C*03:02* | 1.01 | 0.80 | 1 | 1 |  | 1.03 | 0.87 |  | 0.96 | 0.89 |
| *C*03:04* | 1.19 | 0.26 | 0.22 | 0.24 |  | 1.14 | 0.41 |  | 1.09 | 0.65 |
| *C*07:02* | 1.07 | 4.3E-2 | 0.58 | 0.57 |  | 1.19 | 0.19 |  | 1.17 | 0.30 |
| *C*08:01* | 0.86 | 0.16 | 0.40 | 0.39 |  | 0.90 | 0.59 |  | 0.84 | 0.40 |
| *DPB1*02:01* | 0.81 | 9.8E-2 | 8.7E-2 | 7.8E-2 |  | 0.82 | 0.17 |  | 0.92 | 0.60 |
| *DPB1*02:02* | 0.83 | 0.31 | 0.28 | 0.26 |  | 0.83 | 0.30 |  | 0.79 | 0.24 |
| *DPB1*04:01* | 1.01 | 0.63 | 1 | 1 |  | 0.98 | 0.93 |  | 0.97 | 0.92 |
| ***DPB1*05:01*** | **1.42** | **3.0E-7** | **1.0E-4** | **1.0E-5** |  | **2.34** | **4.7E-8** |  | **2.43** | **7.7E-8** |
| *DQB1*02:01* | 1.22 | 0.25 | 0.22 | 0.22 |  | 1.19 | 0.32 |  | 1.15 | 0.47 |
| *DQB1*03:01* | 0.84 | 5.1E-3 | 0.12 | 0.11 |  | 0.92 | 0.55 |  | 0.88 | 0.40 |
| ***DQB1*03:02*** | **0.64** | **1.6E-2** | **1.2E-2** | **1.0E-2** |  | **0.62** | **8.9E-3** |  | **0.65** | **3.1E-2** |
| *DQB1*03:03* | 0.94 | 0.78 | 0.63 | 0.64 |  | 0.96 | 0.78 |  | 1.02 | 0.95 |
| *DQB1*04:01* | 0.93 | 0.75 | 0.72 | 0.72 |  | 0.96 | 0.85 |  | 0.96 | 0.91 |
| ***DQB1*05:02*** | **1.89** | **3.0E-5** | **2.9E-6** | **1.0E-5** |  | **2.01** | **4.3E-6** |  | **2.00** | **1.9E-5** |
| *DQB1*06:01* | 0.98 | 0.44 | 0.88 | 0.89 |  | 0.93 | 0.63 |  | 0.87 | 0.44 |
| *DRB1*03:01* | 1.17 | 0.40 | 0.32 | 0.31 |  | 1.15 | 0.41 |  | 1.13 | 0.55 |
| *DRB1*04:05* | 0.87 | 0.44 | 0.49 | 0.49 |  | 0.90 | 0.58 |  | 0.89 | 0.63 |
| *DRB1*08:03* | 0.84 | 4.2E-2 | 0.32 | 0.34 |  | 0.75 | 0.12 |  | 0.67 | 4.4E-2 |
| *DRB1*09:01* | 0.97 | 0.96 | 0.86 | 0.86 |  | 0.98 | 0.94 |  | 1.05 | 0.79 |
| *DRB1*11:01* | 0.99 | 0.87 | 1 | 1 |  | 0.97 | 0.93 |  | 0.93 | 0.74 |
| ***DRB1*12:02*** | **0.53** | **1.1E-3** | **6.0E-4** | **5.6E-4** |  | **0.51** | **4.9E-4** |  | **0.54** | **3.5E-3** |
| ***DRB1*15:01*** | **1.58** | **6.3E-3** | **5.0E-3** | **4.6E-3** |  | **1.68** | **2.8E-3** |  | **1.84** | **1.3E-3** |
| ***DRB1*16:02*** | **2.44** | **8.9E-7** | **3.5E-7** | **1.9E-7** |  | **2.63** | **1.6E-7** |  | **2.74** | **3.0E-7** |

^a^ OR, odds ratio.

^b^ The 8 alleles in boldface had nominal Cochran-Armitage trend test *P* value smaller than 0.05.
