# Supplementary material for: Comprehensive Genotyping in Two Homogeneous Graves' Disease Samples Reveals Major and Novel HLA Association Alleles
Source: PLoS One. 2011 Jan 28;6(1):e16635. doi: 10.1371/journal.pone.0016635 (PMC3030609; doi:10.1371/journal.pone.0016635)
Supplement: Table S4 — Association results of family-based association test. (DOC) [file pone.0016635.s004.doc]

**Table S4.** Association results of family-based association test.

| **HLA allele** | **Allele frequency^a^** | **Number of families** | **FBAT z score^b^** | **FBAT nominal *P* value** |
| --- | --- | --- | --- | --- |
| *A*01:01* | 0.008 | 4 | -1.897 | 0.0578 |
| *A*01:02* | 0.002 | 1 | 1.000 | 0.3173 |
| *A*02:01/07*^c^ | 0.244 | 74 | 1.520 | 0.1285 |
| *A*02:03* | 0.051 | 24 | 0.123 | 0.9022 |
| *A*02:06* | 0.028 | 13 | 1.080 | 0.2803 |
| *A*03:01* | 0.005 | 3 | 1.000 | 0.3173 |
| ***A*11:01/02*^d^** | **0.336** | **74** | **1.126** | **0.2601** |
| *A*24:02* | 0.17 | 65 | -1.667 | 0.0956 |
| *A*24:03* | - | - | - | - |
| *A*26:01* | 0.028 | 17 | -1.273 | 0.2029 |
| *A*30:01* | 0.009 | 5 | -0.420 | 0.6745 |
| *A*31:01* | 0.014 | 9 | -1.056 | 0.2908 |
| *A*32:01* | 0.003 | 2 | -1.342 | 0.1797 |
| *A*33:03* | 0.097 | 42 | -0.060 | 0.9519 |
| *A*34:01* | 0.002 | 1 | -1.000 | 0.3173 |
| *A*68:01* | 0.003 | 2 | -0.277 | 0.7815 |
| *B*07:02* | 0.002 | 1 | -1.000 | 0.3173 |
| *B*07:05* | 0.005 | 1 | -1.000 | 0.3173 |
| *B*08:01* | 0.003 | 2 | 1.414 | 0.1573 |
| *B*13:01* | 0.077 | 35 | 0.635 | 0.5251 |
| *B*13:02* | 0.008 | 4 | -0.238 | 0.8122 |
| *B*15:01* | 0.026 | 17 | -2.184 | 0.0290 |
| *B*15:02* | 0.03 | 15 | -1.098 | 0.2722 |
| *B*15:11* | 0.006 | 2 | 0.508 | 0.6117 |
| *B*15:12* | 0.003 | 1 | 1.000 | 0.3173 |
| *B*15:18* | 0.005 | 2 | 0.447 | 0.6547 |
| *B*15:25* | 0.006 | 3 | -1.671 | 0.0947 |
| *B*15:27* | 0.008 | 4 | -1.387 | 0.1655 |
| *B*15:03/07/32* | 0.005 | 2 | -0.707 | 0.4794 |
| *B*27:04/05* | 0.03 | 11 | 0.106 | 0.9156 |
| *B*35:01* | 0.037 | 18 | 0.165 | 0.8692 |
| *B*35:03* | 0.005 | 3 | 0.816 | 0.4142 |
| *B*37:01* | 0.006 | 3 | -1.633 | 0.1020 |
| *B*38:01/02* | 0.033 | 18 | -0.647 | 0.5175 |
| *B*39:01/05* | 0.026 | 11 | -0.784 | 0.4328 |
| *B*39:15* | 0.002 | 2 | 1.414 | 0.1573 |
| *B*40:01/48* | 0.216 | 72 | 0.812 | 0.4169 |
| *B*40:02/03* | 0.013 | 9 | -2.197 | 0.0280 |
| *B*40:06* | 0.017 | 7 | -0.393 | 0.6942 |
| *B*44:02* | 0.003 | 1 | -1.000 | 0.3173 |
| ***B*46:01*** | **0.174** | **68** | **2.778** | **0.0055** |
| *B*48:01/03* | 0.013 | 4 | 1.134 | 0.2568 |
| *B*51:01/02* | 0.051 | 25 | 0.821 | 0.4119 |
| *B*52:01* | 0.006 | 3 | -0.816 | 0.4142 |
| *B*54:01/02* | 0.032 | 12 | -0.069 | 0.9451 |
| *B*55:01/02* | 0.037 | 19 | -0.662 | 0.5078 |
| *B*56:01/03/04* | 0.011 | 5 | 0.905 | 0.3657 |
| *B*57:01* | 0.005 | 2 | -1.342 | 0.1797 |
| *B*58:01* | 0.099 | 43 | 0.072 | 0.9429 |
| *C*01:02* | 0.232 | 71 | 2.477 | 0.0132 |
| *C*01:02/03* | 0.009 | 3 | -1.671 | 0.0947 |
| *C*01:03* | 0.008 | 5 | 0.905 | 0.3657 |
| *C*02:02* | 0.002 | 1 | 1.000 | 0.3173 |
| *C*03:02* | 0.102 | 44 | 0.298 | 0.7654 |
| *C*03:03* | 0.046 | 21 | -1.260 | 0.2077 |
| *C*03:03/04* | 0.003 | 2 | -1.414 | 0.1573 |
| *C*03:04* | 0.113 | 46 | -1.371 | 0.1703 |
| *C*04:01* | 0.03 | 19 | -1.769 | 0.0769 |
| *C*04:03* | 0.018 | 9 | 0.124 | 0.9013 |
| *C*05:01* | 0.003 | 1 | -1.000 | 0.3173 |
| *C*06:02* | 0.018 | 8 | -1.326 | 0.1849 |
| *C*07:02* | 0.219 | 81 | 0.863 | 0.3882 |
| *C*07:04* | 0.005 | 2 | 0.447 | 0.6547 |
| *C*07:14* | 0 | 1 | -1.000 | 0.3173 |
| *C*08:01* | 0.068 | 24 | -1.133 | 0.2573 |
| *C*08:03* | 0.003 | 1 | 1.000 | 0.3173 |
| *C*12:02* | 0.046 | 18 | 0.374 | 0.7084 |
| *C*12:03* | 0.014 | 7 | 0.832 | 0.4054 |
| *C*14:02* | 0.038 | 20 | 0.034 | 0.9729 |
| *C*15:02* | 0.022 | 14 | 0.633 | 0.5267 |
| *C*16:02* | 0.002 | 1 | -1.000 | 0.3173 |
| *C*16:04* | - | - | - | - |
| *DPB1*01:01* | 0.002 | 1 | -1.000 | 0.3173 |
| *DPB1*02:01* | 0.13 | 55 | -0.413 | 0.6797 |
| *DPB1*02:02* | 0.09 | 45 | 0.179 | 0.8582 |
| *DPB1*03:01* | 0.045 | 25 | -1.779 | 0.0752 |
| *DPB1*04:01* | 0.079 | 42 | 1.376 | 0.1689 |
| *DPB1*04:02* | 0.015 | 7 | -1.137 | 0.2557 |
| ***DPB1*05:01*** | **0.51** | **71** | **3.966** | **0.000073** |
| *DPB1*09:01* | 0.014 | 7 | -2.164 | 0.0304 |
| *DPB1*13:01* | 0.054 | 28 | -1.461 | 0.1441 |
| *DPB1*14:01* | 0.026 | 9 | -2.069 | 0.0386 |
| *DPB1*16:01* | 0.002 | 1 | -1.000 | 0.3173 |
| *DPB1*17:01* | 0.003 | 2 | 0.061 | 0.9517 |
| *DPB1*19:01* | 0.003 | 2 | -1.342 | 0.1797 |
| *DPB1*21:01* | 0.015 | 9 | 0.000 | 1.0000 |
| *DPB1*22:01* | 0.003 | 2 | -1.414 | 0.1573 |
| *DPB1*31:01* | 0.002 | 1 | 1.000 | 0.3173 |
| *DPB1*36:01* | 0.002 | 1 | -1.000 | 0.3173 |
| *DPB1*41:01* | 0.002 | 0 | - | - |
| *DPB1*100:01* | 0.002 | 1 | -1.000 | 0.3173 |
| *DPB1*109:01* | 0.002 | 0 | - | - |
| *DQB1*02:01* | 0.09 | 46 | 0.847 | 0.3969 |
| *DQB1*03:01* | 0.192 | 69 | 1.712 | 0.0869 |
| ***DQB1*03:02*** | **0.065** | **33** | **-1.990** | **0.0466** |
| *DQB1*03:03* | 0.199 | 78 | -0.756 | 0.4499 |
| *DQB1*04:01* | 0.072 | 27 | -0.749 | 0.4536 |
| *DQB1*04:02* | 0.013 | 6 | -0.618 | 0.5367 |
| *DQB1*05:01* | 0.013 | 8 | -0.535 | 0.5930 |
| ***DQB1*05:02*** | **0.102** | **45** | **-0.252** | **0.8010** |
| *DQB1*05:03* | 0.046 | 22 | -0.739 | 0.4602 |
| *DQB1*06:01* | 0.13 | 60 | 0.999 | 0.3180 |
| *DQB1*06:02* | 0.055 | 31 | 0.952 | 0.3413 |
| *DQB1*06:03* | 0.005 | 2 | -1.414 | 0.1573 |
| *DQB1*06:04* | 0.002 | 1 | 1.000 | 0.3173 |
| *DQB1*06:09* | 0.014 | 6 | -2.248 | 0.0246 |
| *DQB1*06:10* | 0.003 | 2 | 0.000 | 1.0000 |
| *DQB1*06:11* | 0 | 1 | -1.000 | 0.3173 |
| *DRB1*01:01* | 0.005 | 3 | 0.577 | 0.5637 |
| *DRB1*03:01* | 0.084 | 43 | 0.881 | 0.3785 |
| *DRB1*04:01* | 0.003 | 2 | 0.447 | 0.6547 |
| *DRB1*04:03* | 0.026 | 13 | -0.756 | 0.4497 |
| *DRB1*04:04* | 0.005 | 2 | 0.563 | 0.5734 |
| *DRB1*04:05* | 0.072 | 26 | -0.896 | 0.3702 |
| *DRB1*04:06* | 0.031 | 17 | -1.687 | 0.0917 |
| *DRB1*04:10* | 0.003 | 1 | -1.000 | 0.3173 |
| *DRB1*07:01* | 0.011 | 6 | -1.414 | 0.1573 |
| *DRB1*08:02* | 0.006 | 3 | 1.732 | 0.0833 |
| *DRB1*08:03* | 0.091 | 41 | -0.197 | 0.8440 |
| *DRB1*08:09* | 0.005 | 2 | -1.412 | 0.1579 |
| *DRB1*09:01* | 0.191 | 74 | -0.189 | 0.8498 |
| *DRB1*09:02* | 0.002 | 1 | -1.000 | 0.3173 |
| *DRB1*10:01* | 0.005 | 3 | -1.633 | 0.1025 |
| *DRB1*11:01* | 0.074 | 34 | 0.677 | 0.4983 |
| *DRB1*11:04* | - | - | - | - |
| *DRB1*12:01* | 0.045 | 25 | 1.146 | 0.2516 |
| ***DRB1*12:02*** | **0.065** | **31** | **-0.500** | **0.6170** |
| *DRB1*13:01* | 0.005 | 2 | -1.414 | 0.1573 |
| *DRB1*13:02* | 0.018 | 7 | -2.086 | 0.0370 |
| *DRB1*13:12* | 0.008 | 1 | 1.000 | 0.3173 |
| *DRB1*14:01* | 0.029 | 15 | -1.450 | 0.1472 |
| *DRB1*14:02* | 0 | 1 | 1.000 | 0.3173 |
| *DRB1*14:03* | 0.003 | 3 | 0.000 | 1.0000 |
| *DRB1*14:04* | 0.003 | 1 | 1.000 | 0.3173 |
| *DRB1*14:05* | 0.029 | 12 | -0.302 | 0.7630 |
| *DRB1*14:35* | - | - | - | - |
| ***DRB1*15:01*** | **0.118** | **57** | **1.972** | **0.0486** |
| *DRB1*15:02* | 0.01 | 6 | -0.626 | 0.5316 |
| ***DRB1*16:02*** | **0.054** | **28** | **0.452** | **0.6516** |

^a^ Allele frequencies calculated based on family founders.

^b^ Family-based association test (FBAT) under dominant model. The option “-e” was used to test association in the presence of linkage. A value smaller than zero indicates the allele is “under-transmitted” to the affected individuals.

^c^ Among all the rows listed as the combination of at least two alleles, 4 of them (*A*02:01/07*, *A*11:01/02*, *C*01:02/03* and *C*03:03/04*) were caused by the limitation of genotyping resolution (i.e. the genotyping kit could not separate these alleles); the others were combined together because that the FBAT software package had its maximal number of allowed alleles. We only combined those alleles with relatively low frequencies, which did not affect the association signals we reported here in the manuscript.

^d^ The 8 alleles in boldface were the alleles with best *P* values in our case-control study
